# A scoring model for differentiating gastric calcifying fibrous tumors from gastrointestinal stromal tumors less than 2 cm based on CT features

**DOI:** 10.1186/s12880-026-02322-2

**Published:** 2026-03-31

**Authors:** Huijia Yin, Jianxia Xu, Beiran Wang, Dan Zang, Shuaina Wang, Sujuan Liu, Hongpo Wang, Dongming Han, Risheng Yu

**Affiliations:** 1 Department of MR, The First Affiliated Hospital of Xinxiang Medical University, Weihui, 453100 China; 2 Department of Radiology, The Second Affiliated Hospital of Zhejiang Chinese Medical University, Hangzhou, 310005 China; 3 Department of Pathology, The First Affiliated Hospital of Xinxiang Medical University, Weihui, 453100 China; 4 Department of Radiology, The Second Affiliated Hospital, Zhejiang University School of Medicine, Hangzhou, 310009 China

**Keywords:** Calcifying fibrous tumors, Gastrointestinal stromal tumors, CT features, Scoring model

## Abstract

**Objectives:**

This study aimed to compare the CT features of calcifying fibrous tumors (CFTs) and gastrointestinal stromal tumors (GISTs) less than 2 cm, and to establish a scoring system to differentiate them.

**Methods:**

A total of 85 patients were included, comprising 22 CFTs and 63 GISTs. Clinical and CT imaging features were compared between the two groups. Independent predictors were identified using univariate and multivariate logistic regression analyses. A predictive model was constructed and converted into a simplified scoring system based on regression coefficients. Model performance was evaluated using receiver operating characteristic (ROC) analysis and the Hosmer–Lemeshow goodness-of-fit test.

**Results:**

The analysis revealed eight variables with statistically significant differences (*P* < 0.05). Four variables were included in the score model, including long diameter/short diameter (LD/SD) ratio, calcification (mild), calcification (moderate), and degree of enhancement (moderate), and identified as independent predictors of CFT. The scoring system ranged from − 2 to 5 points, where higher scores correlated with a greater likelihood of CFT. The AUCs for the predictive model and scoring model were 0.833 and 0.822, respectively, with no significant difference between the two (*P* = 0.595). To facilitate clinical application, the scoring system was divided into four ranges, with corresponding probabilities of CFT of 7.14%, 14.29%, 64.71%, and 80.00%.

**Conclusion:**

The proposed CT-based predictive model and scoring system demonstrate good diagnostic performance and may enhance diagnostic confidence, potentially reducing unnecessary invasive procedures when CFT is suspected.

**Supplementary Information:**

The online version contains supplementary material available at 10.1186/s12880-026-02322-2.

## Introduction

Calcifying fibrous tumors (CFTs) are rare benign mesenchymal tumors of the gastrointestinal tract, which was first described by Rosenthal et al. [1]in 1988. CFTs are most commonly discovered incidentally without clinical symptoms [2–4]. CFTs have traditionally been considered soft tissue tumors and have been reported in various anatomical locations, including the pleura, mediastinum, spine, limbs, heart, etc [3, 5]. However, CFTs of the gastrointestinal tract have only recently been gradually reported [3, 6–8]. These findings have improved clinical recognition and suggest that the majority of these tumors may actually occur in the gastrointestinal tract [3, 9].

The tumor may be completely asymptomatic or may cause mild discomfort in the upper abdomen. CFT is histologically characterized by paucicellular collagen matrix, interspersed calcified bodies, and a sparse inflammatory infiltrate [10]. The calcified component, may be either psammomatous or dystrophic [11]. Therefore, CFT is classified as benign tumor. The characteristics of gastric CFT are different from GIST. However, CFTs are frequently misdiagnosed as gastrointestinal stromal tumors (GISTs) in clinical practice [3], primarily due to a lack of familiarity with the condition. This misdiagnosis is particularly common for tumors smaller than 2 cm, which are among the most frequently encountered mesenchymal tumors of the stomach. Small gastric subepithelial tumors measuring less than 2 cm are clinically important because both benign lesions and low-risk GISTs commonly fall within this size range, potentially leading to different follow-up and treatment approaches [12]. Other potential differential diagnoses include leiomyoma, schwannoma, solitary fibrous tumor, and inflammatory myofibroblastic tumor, among others [13, 14].

The treatment and prognosis of CFTs differ significantly from those of GISTs. When endoscopic submucosal dissection (ESD) is considered, confirmation that the lesion arises from the mucosal or submucosal layer is an essential prerequisite. Lesions originating from the muscularis propria are generally not suitable for ESD. For small and asymptomatic gastric CFTs, long-term surveillance or endoscopic submucosal dissection(ESD) is considered sufficient [15]. However, GISTs exhibit variable malignant potential. While GISTs with low malignant potential and a size less than 2 cm can be either resected or closely monitored [16]. They have the potential to enlarge over time and, in some cases, undergo malignant transformation. Thus, regular monitoring and risk assessment are essential. Also, the differentiation between CFT and GIST is of great significance. So far, there have been few studies on CFTs, mostly reviews and case report [17–20]. Only one original study has directly compared the CT features of gastric CFTs and GISTs, reporting differences in tumor location, enhancement patterns, and attenuation values [21]. However, no quantitative predictive model or scoring system has been developed.

Therefore, this retrospective, two-center study aims to establish a scorin*g* system by analyzing the clinical and imaging characteristics of gastric CFTs and GISTs less than 2 cm, to identify discriminative variables, and to construct a predictive model to support imaging-based differentiation between these two entities.

## Materials and methods

### Patients

**Fig. 1:**
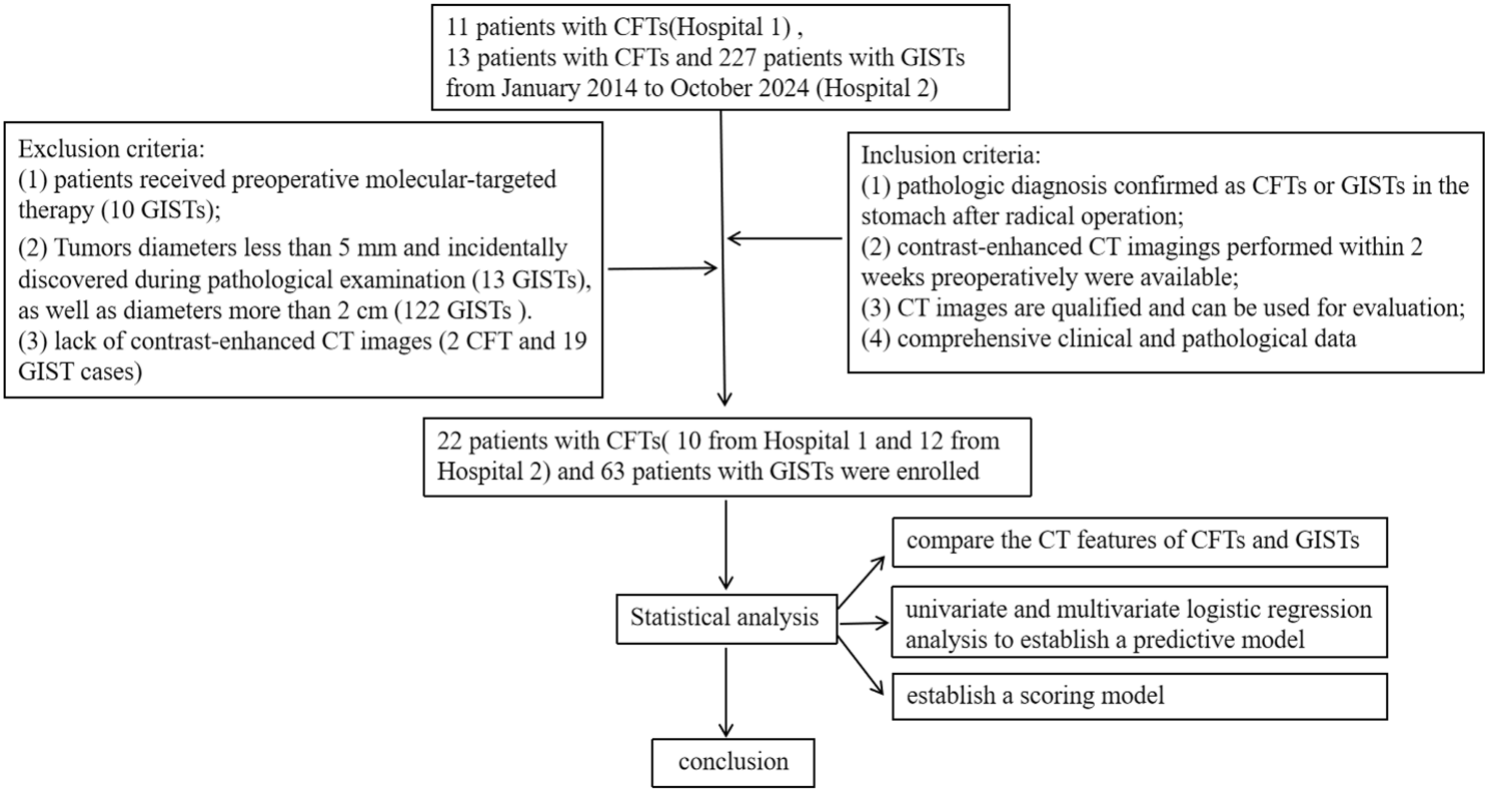
Flow of chart

Our study population was obtained from two independent hospitals: the First Affiliated Hospital of Xinxiang Medical University (hospital 1) and the Second Affiliated Hospital, Zhejiang University School of Medicine (hospital 2). The institutional review boards of these two hospitals approved this retrospective study and informed consent was waived for this type of study design. Initially, a total of 251 patients were discovered, including 13 patients with CFTs and 227 patients with GISTs from hospital 2 and 11 patients with CFTs from hospital 1. The flowchart of this study is shown in Fig. 1.

The inclusion criteria were as follows: (1) pathologic diagnosis confirmed as CFTs or GISTs in the stomach after radical operation; (2) contrast-enhanced CT imagings performed within 2 weeks preoperatively were available; (3) CT images were of adequate quality for evaluation; 4) comprehensive clinical and pathological data. In total, 2 CFT patients and 164 GIST patients were excluded. The exclusion criteria were as follows: (1) patients who had received preoperative molecular-targeted therapy (10 GIST patients); (2) tumors diameters less than 5 mm and incidentally discovered during pathological examination (13 GIST patients), as well as diameters more than 2 cm (122 GIST patients). Additionally, 21 patients were excluded for lack of contrast-enhanced CT images (2 CFT and 19 GIST cases). Overall, 22 patients with gastric CFTs (10 from hospital 1 and 12 from hospital 2) and 63 patients with gastric GISTs (all from hospital 2) were enrolled in this retrospective study.

### Imaging acquisition

Abdominal contrast-enhanced CT examinations in hospital 1 were performed on a 320-slice CT scanner (Aquilion One, Toshiba Medical Systems, Otawara, Japan). Contrast-enhanced CT in hospital 2 was performed using Multidetector -row CT.

(SOMATOM Definition Flash; Siemens Healthcare, Erlangen, Germany). All enrolled patients underwent non-contrast and contrast-enhanced CT scans. Patient preparation before examination was as follows: fast for at least 6 h before CT examination; injected 10 mg of anisodamine intramuscularly 10 min before scanning to reduce peristalsis of the gastrointestinal tract; drunk 800–1000 ml water after injection to expand the stomach fully. Patients were examined in the supine position, and the scanning range was from the diaphragm dome to the pubic symphysis. The CT scanning parameters were as follows throughout: detector configuration 128 × 0.6 mm, tube voltage 120 kVp, tube current 200 mAs, slice thickness 5 mm, slice interval 5 mm, pitch 0.6 mm. The contrast agents in the two hospitals were Ultravist (Bayer Schering Pharma, Berlin, Germany) and Optiray (Liebel-Flarsheim Canada Inc., Kirkland, Quebec., Canada), respectively. A total of 100 ml of iodinated contrast agent was administered via a pump injector at a rate of 5 ml/s into an antecubital vein. The arterial phase, portal venous phase and the equilibrium phase scans were performed at 7–12 s, 50–60 s and 100–110 s after contrast medium injection, respectively.

### Imaging evaluation

Two abdominal radiologists (with 6 and 14 years of clinical experience respectively), who were blinded to the pathological results, reviewed CT images of all patients independently. If there were any disagreements between the two observers, a third experienced abdominal radiologist with more than 30 years of experience was invited to resolve. Images were reviewed on a picture archive communication system.

The following qualitative imaging parameters of the lesions were evaluated: (1) tumor location (cardia, fundus, body, or antrum of stomach). (2) margin (regular, irregular). Smooth margins were defined as regular, and lobulated margins were defined as irregular. (3) border (well-defined, ill-defined). A distinct tumor border was considered well-defined. If presented with surrounding fat infiltration or had an unclear border, the tumor was considered ill-defined. (4) contour (round-like, elliptical, lobulated). This feature was evaluated on the three-dimensional reconstruction image. (5) growth pattern (endoluminal, mixed, exophytic). Depending on whether more than 95% of the lesion is inside the cavity or not. If inside the cavity, it is the endoluminal type; if outside the cavity, it is the exophytic type; otherwise it is a mixed type. (6) degrees of calcification (none, mild, moderate, diffuse). Calcification was defined as a high-attenuation region, that is, CT value > 100HU in the unenhanced stage. Mild is limited. Moderate is calcification areas < 50% of the lesion, excluding punctate calcification and diffuse calcification (calcification areas > 50% of the lesion). (7) enhancement pattern (homogeneous, heterogeneous). Homogeneous enhancement indicated that the difference between the most strongly and weakly enhanced portion of the lesions was less than 10 HU, or indicated heterogeneous enhancement. (8) degree of enhancement. The degree of enhancement was quantitatively defined according to the difference between the post-enhancement CT attenuation value and the non-enhanced CT attenuation value. If the difference was < 20 HU, the tumor was considered to exhibit a low-mild enhancement pattern; 20–40 HU was considered to represent a moderate enhancement pattern, and > 40 HU was judged to be a strong enhancement pattern.

The quantitative image analysis were as follows: (1) the long diameter (LD) and short diameter (SD) of each lesions. The measurement were performed through multiplanar reformation (axial, coronal, and sagittal), and the LD/SD ratios were calculated. (2) CT values of non-enhanced scan, arterial phase, the portal venous phase, and equilibrium phase enhancement. The region-of-interest (ROI) were placed on the parenchyma in all lesions using a 20–30 mm^2^ circular, CT attenuation values of each phase were measured in HU. Then the differences were calculated, including difference value 1 (the arterial phase values minus the non-enhanced phase values), difference value 2 (the portal venous phase values minus the non-enhanced phase values), difference value 3 (the equilibrium phase CT values minus the non-enhanced phase values), and difference value 4 (the equilibrium phase CT values minus the portal venous phase values).

### Statistical analysis

All data were analyzed using SPSS 26.0 (IBM SPSS Statistics, Armonk, NY) and MedCalc 22.0 (MedCalc, Mariakerke, Belgium). The normality of quantitative variables was assessed using the Kolmogorov–Smirnov test. Normally distributed variables are presented as mean ± standard deviation, while non-normally distributed variables are presented as median (P25, P75). Categorical variables are expressed as frequencies (percentages). First, the clinical and imaging characteristics of CFT and GIST patients were compared. The independent sample t-test was applied to normally distributed quantitative variables, while the Mann–Whitney U test was used for non-normally distributed variables. Categorical variables were compared using the Chi-square test or Fisher’s exact test, as appropriate. The reproducibility of quantitative features was assessed using the intraclass correlation coefficient (ICC), with an ICC greater than 0.75 indicating good consistency [22].The consistency of categorical variables was assessed using the Cohen’s kappa coefficient. A Kappa value greater than 0.81 indicates near-perfect consistency, 0.61–0.80 represents substantial consistency, 0.41–0.60 indicates moderate consistency, 0.21–0.40 signifies fair consistency, and a Kappa value less than 0.20 reflects slight consistency [23].

Variables that did not show variability across all cases (e.g., margin, ulceration, hemorrhage, necrosis, and intratumoral vessels, where all patients were negative) were excluded from the analysis due to their lack of statistical discriminative power. After univariate analysis, variables with P value < 0.05 were included in the multivariate analysis. Multivariate analysis was performed using Stepwise logistic regression (backward stepwise logistic regression), which iteratively eliminated non-significant variables to identify independent predictors for constructing the final predictive model. To establish a scoring system that is easy to calculate, regression coefficients were transformed into weighted integer scores. Each coefficient (β_i_) was divided by half of the smallest absolute coefficient and rounded to the nearest integer to derive the corresponding weight (w_i_). The total score for each patient was calculated as the sum of the weighted predictors (Score = w₁X₁ + w₂X₂ + … + wₙXₙ), where w_i_ represents the assigned integer weight for each independent predictor and X_i_ represents the coded value of the corresponding predictor as defined in the regression model [24]. During the model construction process, multicollinearity among variables was assessed using the variance inflation factor (VIF) and tolerance values, with a VIF > 10 indicating the presence of multicollinearity. The goodness-of-fit of the model was evaluated using the Hosmer-Lemeshow test, where P value > 0.05 suggests a good model fit. Subsequently, the area under the receiver operating characteristic (ROC) curve (AUC) was calculated to assess the discriminative ability of the model, and the DeLong test was used to compare ROC curves between different models. P value < 0.05 was considered statistically significant in all statistical tests.

## Results

### Clinical baseline characteristics

**Fig. 2:**
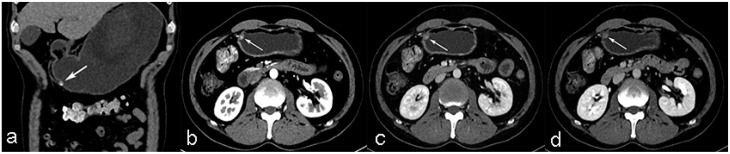
Gastric calcifying fibrous tumor (CFT) **a**. unenhanced phase coronal CT image shows an elliptical, soft tissue lesion in antrum of stomach with moderate calcification (arrow). **b**, **c** and **d**, arterial phase, portal venous phase and equilibrium phase of CT images show moderate enhancement

**Fig. 3:**
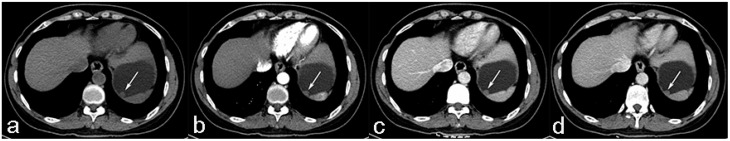
Gastrointestinal stromal tumor (GIST) **a**. unenhanced phase coronal CT image shows a round-like, soft tissue lesion in fundus of stomach without calcification (arrow). **b**, **c** and **d**, arterial phase, portal venous phase and equilibrium phase of CT images show mild enhancement

A total of 85 patients were included in our study, comprising 22 cases of CFT(Fig. 2) and 63 cases of GIST(Fig. 3). The clinical baseline characteristics of the two groups are summarized in Table 1. The mean age of the patients with CFT was significantly lower than that of the GIST group (48.05 ± 10.89 years vs. 59.21 ± 9.02 years, *P* < 0.001). In terms of sex distribution, there was no significant difference between the two groups (*P* = 0.356). Smoking history showed a significant difference, with 22.73% of CFT patients and only 6.35% of GIST patients reporting smoking (*P* = 0.046). No significant differences were observed in drinking history (*P* = 1.000) or the presence of associated symptoms (*P* = 0.734) between the two groups.

**Table 1:** Clinical baseline characteristics

Parameters	CFT(*n* = 22)	GIST(*n* = 63)	*P* value
Age(years)	48.05 ± 10.89	59.21 ± 9.02	<0.001
Sex			0.356
Male	9(40.90%)	19(30.16%)	
Female	13(59.10%)	44(69.84%)	
Smoke			0.046
Yes	5(22.73%)	4(6.35%)	
No	17(77.27%)	59(93.65%)	
Drink			1.000
Yes	1(4.54%)	4(6.35%)	
No	21(95.45%)	59(93.65%)	
Associated symptoms			0.734
Yes	18(81.82%)	9(14.29%)	
No	4(18.18%)	54(85.71%)	

CFT: Calcifying fibrous tumor; GIST: Gastrointestinal stromal tumor

### Inter-observer agreement

The inter-observer agreement between the two readers was assessed using both Intraclass Correlation Coefficients (ICC) and Cohen’s Kappa for various imaging features. The ICC values for the CT phase assessments were as follows: 0.911 (95% CI: 0.864–0.942) for non-contrast CT, 0.909 (95% CI: 0.863–0.940) for arterial phase CT, 0.885 (95% CI: 0.828–0.923) for venous phase CT, and 0.927 (95% CI: 0.891–0.952) for delayed phase CT. The ICC for the LD/SD ratio was 0.815 (95% CI: 0.726–0.876), all indicating good to excellent consistency with significant P-values (*P* < 0.05). For categorical features, kappa values were 0.864 for growth pattern, 0.821 for margin, 0.805 for contour, 0.713 for calcification, and 0.691 for pattern of enhancement (all *P* < 0.05), demonstrating substantial to near-perfect agreement. Weighted kappa for degree of enhancement was 0.726 (*P* < 0.05). Kappa values for border, surface ulceration, hemorrhage, necrosis, and intratumoral vessels were not calculable due to lack of variability.

### Comparisons of CT features between CFT and GIST

We performed a comparative analysis of the CT features of CFT and GIST to identify significant imaging differences between the two groups. The results revealed that eight variables revealed statistically significant differences between the groups (*P* < 0.05), including calcification, enhancement degree, LD/SD ratio, non-contrast CT value, arterial phase CT value, delayed phase CT value, and the difference values (Difference value 1 and Difference value 2) (Table 2).

**Table 2:** Comparison of imaging features between CFT and GIST: quantitative and categorical variables

CT features	CFT(*n* = 22)	GIST(*n* = 63)	*P* value
Location			0.112
Fundus	5 (22.73%)	29(46.03%)	
Body	17(77.27%)	31(49.21%)	
Cardia	0 (0.00%)	2 (3.17%)	
Antrum	0 (0.00%)	1 (1.59%)	
Margin			1.000
Irregular	1 (4.54%)	5 (7.94%)	
Regular	21 (95.45%)	58 (92.06%)	
Growth pattern			1.000
Intraluminal	14 (63.64%)	41 (65.08%)	
Extraluminal	4 (18.18%)	12 (19.05%)	
Mixed	4 (18.18%)	10 (15.87%)	
Border			
Ill-defined	0 (0.00%)	0 (0.00%)	
Well-defined	22 (100.00%)	63 (100.00%)	
Contour			0.399
Round-like	5 (22.73%)	24 (38.10%)	
Elliptical	16 (72.73%)	36 (57.14%)	
Lobulated	1 (4.54%)	3 (4.76%)	
Calcification			0.006
None	9 (40.91%)	49 (77.78%)	
Mild	4 (18.18%)	4 (6.35%)	
Moderate	7 (31.82%)	6 (9.52%)	
Diffuse	2 (9.09%)	4 (6.35%)	
Surface ulceration			
Present	0 (0.00%)	0 (0.00%)	
Absent	22 (100.00%)	63 (100.00%)	
Hemorrhage			
Present	0 (0.00%)	0 (0.00%)	
Absent	22 (100.00%)	63 (100.00%)	
Necrosis			
Present	0 (0.00%)	0 (0.00%)	
Absent	22 (100.00%)	63 (100.00%)	
Intratumoral vessels			
Present	0 (0.00%)	0 (0.00%)	
Absent	22 (100.00%)	63 (100.00%)	
Pattern of enhancement			0.165
Homogeneous	11 (50.00%)	21 (33.33%)	
heterogeneous	11 (50.00%)	42 (66.67%)	
Degree of enhancement			0.036
Mild	10 (45.45%)	12 (19.05%)	
Moderate	7 (31.82%)	37 (58.73%)	
Significant	5 (22.73%)	14 (22.22%)	
LD/SD ratio	1.55 ± 0.29	1.35 ± 0.25	0.002
Non-contrast CT value	65.00 (56.38,69.00)	43.00 (34.00,49.00)	< 0.001
Arterial phase CT value	70.00 (64.75,81.50)	58.00 (50.00,72.00)	0.001
Venous phase CT value	76.00 (62.00,87.00)	79.5 (73.75,88.75)	0.139
Delayed phase CT value	81.00 (69.00,95.00)	96.50 (85.75,123.00)	< 0.001
Difference value 1	9.50 (4.75,17.25)	15.00 (8.00,25.00)	0.040
Difference value 2	15.50 (9.00,24.00)	30.00 (22.00,38.00)	< 0.001
Difference value 3	28.50 (19.00,45.50)	37.00 (29.00,40.00)	0.264
Difference value 4	7.00 (0.75,16.75)	4.00 (1.00,12.00)	0.451

### Development of a predictive model

Univariate logistic regression analysis was initially performed to assess the association of all CT features between the two groups. The analysis identified the following variables as statistically significant (*P* < 0.05): LD/SD ratio, calcification (mild), calcification (moderate), and degree of enhancement (moderate) (Table 3). The variables identified as statistically significant in the univariate analysis were subsequently included in a multivariate logistic regression model to determine independent predictors of CFT. The multivariate analysis demonstrated that LD/SD ratio, calcification (moderate), calcification (diffuse), and degree of enhancement (moderate) were independently and significantly associated with the presence of CFT (Table 4). These four variables were ultimately incorporated into the development of the final predictive model.

**Table 3:** Univariate analysis of imaging features between CFT and GIST

Variables	B	OR (95% CI)	*P* value
Location	0.481	1.618 (0.711–3.681)	0.251
Margin	0.594	1.810 (0.200,16.409)	0.598
Growth pattern			
Intraluminal*			
Extraluminal	-0.024	0.976 (0.270–3.525)	0.971
Mixed	-0.158	1.171 (0.316–4.336)	0.813
Contour			
Round-like*			
Elliptical	0.758	2.133 (0.690–6.599)	0.189
Lobulated	0.470	1.600 (0.137–18.723)	0.708
Calcification			
None*			
Mild	1.695	5.444 (1.147–25.846)	0.033
Moderate	1.849	6.352 (1.728–23.345)	0.005
Diffuse	1.001	2.722 (0.432–17.144)	0.286
Pattern of enhancement	-0.693	0.500 (0.186–1.341)	0.168
Degree of enhancement			
Mild*			
Moderate	-1.483	0.227 (0.071–0.728)	0.013
Significant	-0.847	0.429 (0.114–1.607)	0.209
LD/SD ratio	2.762	15.825 (2.273-110.171)	0.004
Non-contrast CT value	0.000248	1.000 (0.998–1.002)	0.827
Arterial phase CT value	0.000051	1.000 (0.998–1.002)	0.964
Venous phase CT value	-0.000136	1.000 (0.997–1.002)	0.919
Delayed phase CT value	0.000150	1.000 (0.998–1.002)	0.891
Difference value 1	-0.034611	0.966 (0.928–1.006)	0.093
Difference value 2	-0.009071	0.991 (0.977–1.005)	0.204
Difference value 3	-0.005724	0.994 (0.974–1.015)	0.593
Difference value 4	0.001711	1.002 (0.988–1.015)	0.801

*Data were used as the reference variable

**Table 4:** Multivariate regression analysis of CT features and weighted scoring of independent predictors

Variables	B	OR(95% CI)	*P* value	Weighted score
LD/SD ratio	2.413	11.171(1.312–88.780)	0.025	3
Calcification(mild)	2.285	9.826(1.645–62.639)	0.013	3
Calcification(moderate)	2.012	7.476(1.178–30.749)	0.013	2
Calcification(diffuse)	0.824	2.279(0.312–16.631)	0.429	
Degree of enhancement(Moderate)	-1.818	0.162(0.034–0.652)	0.015	-2
Degree of enhancement(Significant)	-1.011	0.364(0.080–1.659)	0.192	

To evaluate potential multicollinearity among the included variables, the variance inflation factor (VIF) and tolerance values were calculated. All variables demonstrated VIF values below the threshold of 10, indicating the absence of multicollinearity. Furthermore, the Hosmer–Lemeshow goodness-of-fit test confirmed that the model exhibited a good level of calibration (*P* = 0.417).

### Development of a scoring model

Based on the results of multivariate analysis, we assigned weighted scores to the CT features to establish a scoring system. Degree of enhancement (Moderate) and Calcification (moderate) were each assigned 2 points; Calcification (mild) was assigned 3 points; LD/SD ratio: < 1.26 (M - SD), 0 points; 1.26 ≤ LD/SD ratio < 1.55 (M), 1 points; 1.55 ≤ LD/SD ratio < 1.84(M + SD), 2 points;≥ 1.84, 3 points. For each patient, the individual scores corresponding to the predictive factors were summed to generate a total score ranging from − 2 to 5 points, referred to as the scoring model. The higher the score, the more likely the lesion is to be a CFT. The Hosmer–Lemeshow goodness-of-fit test indicated that the scoring model had good calibration (*P* = 0.318).

### Comparison of diagnostic performance between the predictive and scoring models

**Fig. 4:**
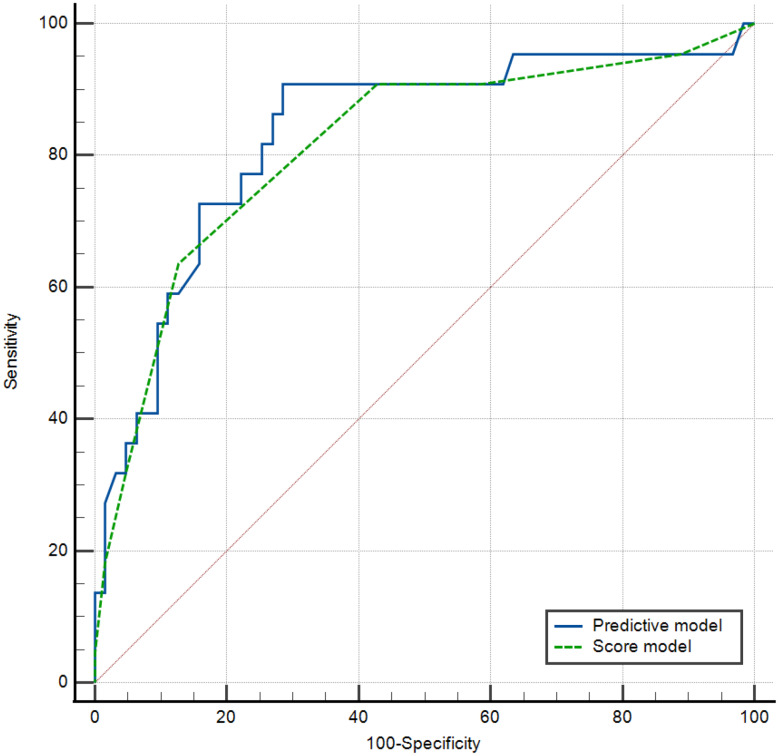
ROC curves of the predictive and scoring models in the diagnosis of CFT

The AUC values for the predictive model and the scoring model were 0.833 (95% CI: 0.736–0.905, *P* < 0.001) and 0.822 (95% CI: 0.724–0.896, *P* < 0.001), respectively (Supplementary Table S1, Fig. 4). The DeLong test was used to compare the AUCs, and the difference between the two models has no statistically significant (95% CI: -0.030–0.052, *P* = 0.595).

### Score ranges exploration

To apply this scoring system conveniently in practice, we further divided it into four score ranges: -2 to -1 points, 0 to 1 points, 2 to 3 points, and 4 to 5 points. Within these score ranges, the probability of CFT in patients is 7.14% for the first range (-2 to -1), 14.29% for the second range (0 to 1 points), 64.71% for the third range (2 to 3 points), and 80.00% for the final range (4 to 5 points) (Table 5).

**Table 5:** Diagnostic probability of CFT in different score ranges (*n* = 85)

Score range	Number of CFT	Total number	Diagnostic probability of CFT
-2 to -1 point	2	28	7.14%
0 to 1 points	5	35	14.29%
2 to 3 points	11	17	64.71%
4 to 5 points	4	5	80.00%

For example, a lesion with an LD/SD ratio of 1.70 (2 points), mild calcification (3 points), and moderate enhancement (− 2 points) would yield a total score of 3 points, corresponding to the 2–3 score range and an estimated CFT probability of 64.71%.

## Discussion

Calcifying fibrous tumor (CFT) is a rare benign tumor, commonly found in the stomach, and is often misdiagnosed as a gastrointestinal stromal tumor (GIST) when smaller than 2 cm [5]. Given the differences in prognosis and management strategies, accurate differentiation between CFT and GIST is clinically important. Small gastric subepithelial tumors measuring ≤ 2 cm are particularly challenging in clinical practice, as imaging features are often subtle and management strategies may differ depending on the presumed diagnosis. To date, only one original study has compared the CT features of gastric CFTs and GISTs [21], reporting differences in conventional CT characteristics such as calcification and enhancement patterns. Our findings are generally consistent with these observations. However, unlike the previous study, we further incorporated the LD/SD ratio into a quantitative scoring system, providing a more structured and objective approach for differentiation. The proposed model, consisting of LD/SD ratio, calcification (mild), calcification (moderate), and degree of enhancement (moderate), showed acceptable discriminative ability for lesions smaller than 2 cm. Although CFTs are frequently misdiagnosed in clinical practice, we did not directly compare the diagnostic performance of the scoring model with that of radiologists without model assistance. Accordingly, the incremental value of the model beyond routine subjective interpretation warrants further investigation.

Our study shows that CFTs mostly located in the fundus and body of the stomach, with regular borders, both intraluminal and extraluminal, all well-defined, mostly elliptical and accompanied by calcification, without ulceration, hemorrhage, necrosis, and intratumoral vessels.

Through comparative analysis, eight parameters were found to be meaningful in distinguishing between the two tumors, which are calcification, enhancement degree, LD/SD ratio, non-contrast CT value, arterial phase CT value, delayed phase CT value, and the difference values. The results are consistent with previous research findings [21]. Calcifications in CFTs perhaps due to psammomatous or dystrophic causes [25]. The higher non-contrast CT values are attributed to the presence of psammomatous bodies and micro calcification in gastric CFTs. However, these variables can only be considered associated factors instead of independent risk or protective factors, for that only univariate analysis was performed. To further statistical verification, multiple regression analyses were performed.

Firstly, we performed univariate analysis to obtain the relevant predictors that were significantly different between CFTs and GISTs. The results revealed four significant features: calcification (mild), calcification (moderate), LD/SD ratio, and degree of enhancement (moderate). In previous study [21], 80% of gastric CFTs exhibited calcification, which is similar to our research findings. A systemic review [18] also indicated that calcification is the main character in the image finding of CFT. The reason may be that calcifications in CFT were psammomatous or dystrophic [3, 11, 26] accompanied by chronic inflammation, mineralization of collagen fibers within fibrous tumors, as well as macrophages may release factors that promote calcification during the process of cleaning damaged tissue [25, 27]. All these factors contribute to the formation of calcification. However, diffuse calcification has no specificity in CFT and is not significantly different between CFT and GIST.

Another important finding of our study is that the LD/SD ratio was significantly higher in CFTs than in GISTs smaller than 2 cm. CFTs tend to present a relatively elongated or elliptical morphology rather than a nearly circular configuration. Quantitative assessment of tumor morphology has been increasingly explored in radiologic research, as shape-related parameters may reflect underlying growth patterns and biological behavior [28, 29]. However, such quantitative morphological analysis has not been applied to gastric CFTs in previous studies [3, 5, 18, 21, 26]. Therefore, incorporating the LD/SD ratio represents a novel aspect of our work. The elongated configuration of CFTs may relate to their tendency to grow along tissue planes and gastric wall boundaries [3, 27], and may also be influenced by the dense collagen matrix and distribution of calcifications.

Another feature, moderate enhancement in CFTs, may be related to its low blood flow, collagen fiber density, and delayed diffusion of contrast agents [6, 30]. Then, multivariate analysis was performed to construct the final predictive model. In CFT, calcification (mild), calcification (moderate), LD/SD ratio, and degree of enhancement (moderate) were independent predictive features, which may assist radiologists in differentiating CFT from small GISTs.

The above four characteristics were finally selected to develop the scoring system through univariate and multivariate regression analyses. The scoring system stratified the model into different scoring ranges to identify CFT. We assign scores to these features in different weights. Among them, the prediction efficiency of 4–5 points is the highest, reaching 80%. Even in the highest score category, the estimated probability of CFT was approximately 80%, indicating that a residual risk of GIST remains. Therefore, the scoring system should not be interpreted as a definitive diagnostic tool, nor should it independently justify changes in management strategy. Our study shows that both the predictive models and score models had good predictive ability for CFTs and GISTs, with AUCs 0.833 and 0.822, respectively. The DeLong test showed that there was no difference between the two models in discriminating of CFT and GIST, suggesting that the score model could effectively replace the predictive model and provide a more convenient evaluation. The above results demonstrate the score model for CFT patients are valuable for personalized medicine.

However, this study has several limitations. First, the small sample size (*n* = 22) and retrospective design may introduce selection bias and reduce statistical power. The low events-per-variable ratio in the multivariable model raises concerns about overfitting. Additionally, the LD/SD ratio was categorized based on sample distribution rather than predefined thresholds, which may limit its interpretability. Second, only GISTs smaller than 2 cm were included, restricting generalizability. The case distribution imbalance between centers, especially the predominance of GIST cases from one center, may introduce center-related bias. Finally, larger multicenter studies, including at least 50 CFT cases with independent validation, are needed.

In conclusion, we analyzed the characteristics in distinguishing CFTs and GISTs, and established an efficient and convenient-to-use scoring system for discriminating CFTs from GISTs less than 2 cm based on CT features. This model may help differentiate gastric CFTs from small GISTs and requires further validation in prospective multicenter studies.

## Supplementary Information

Below is the link to the electronic supplementary material.

Supplementary Material 1

## Data Availability

The datasets used and/or analyzed during the current study are available from the corresponding author on reasonable request.

## Abbreviations

CFT: Calcifying fibrous tumor
GIST: Gastrointestinal stromal tumor
AUC: Area Under the Curve
ESD: Endoscopic submucosal dissection
LD: Long diameter
SD: Short diameter
ROI: Region of interest
ICC: Intraclass correlation coefficient
VIF: Variance inflation factor
