# Supplementary material for: A scoring model for differentiating gastric calcifying fibrous tumors from gastrointestinal stromal tumors less than 2 cm based on CT features
Source: BMC Med Imaging. 2026 Mar 31;26:250. doi: 10.1186/s12880-026-02322-2 (PMC13173732; doi:10.1186/s12880-026-02322-2)
Supplement: Supplementary file 1 — Supplementary Material 1 [file 12880_2026_2322_MOESM1_ESM.docx]

Supplementary Table：

Table S1 Performance metrics of the predictive and scoring models

| **Metric** | **AUC (95% CI)** | **P value** | **Threshold (Cut-off)** | **Youden Index (J)** | **Sensitivity** | **Specificity** |
| --- | --- | --- | --- | --- | --- | --- |
| Predictive model | 0.833 (0.736 - 0.905) | < 0.0001 | > 0.210 | 0.623 | 90.91% | 71.43% |
| Scoring model | 0.822 (0.724 - 0.896) | < 0.0001 | > 1.000 | 0.571 | 68.18% | 88.89% |
